# Serological and clinical outcomes of rituximab in APS with or without SLE: a retrospective single-centre cohort

**DOI:** 10.1093/rheumatology/keag609

**Published:** 2026-10-29

**Authors:** Ömer Uludağ, Gamze Kemec, Soner Altın, Burak İnce, Yasemin Yalçınkaya, Sevil Kamalı, Ahmet Gül, Murat Inanç, Bahar Artim-Esen

**Affiliations:** Division of Rheumatology, Department of Internal Medicine, Istanbul Faculty of Medicine, Istanbul University, Istanbul, Turkey; Division of Rheumatology, Department of Internal Medicine, Istanbul Faculty of Medicine, Istanbul University, Istanbul, Turkey; Department of Internal Medicine, Istanbul Faculty of Medicine, Istanbul University, Istanbul, Turkey; Division of Rheumatology, Department of Internal Medicine, Istanbul Faculty of Medicine, Istanbul University, Istanbul, Turkey; Division of Rheumatology, Department of Internal Medicine, Istanbul Faculty of Medicine, Istanbul University, Istanbul, Turkey; Division of Rheumatology, Department of Internal Medicine, Istanbul Faculty of Medicine, Istanbul University, Istanbul, Turkey; Division of Rheumatology, Department of Internal Medicine, Istanbul Faculty of Medicine, Istanbul University, Istanbul, Turkey; Division of Rheumatology, Department of Internal Medicine, Istanbul Faculty of Medicine, Istanbul University, Istanbul, Turkey; Division of Rheumatology, Department of Internal Medicine, Istanbul Faculty of Medicine, Istanbul University, Istanbul, Turkey

**Keywords:** APS, rituximab, B cell targets, thrombosis, SLE

## Abstract

**Objectives:**

To assess the dynamics of aPL titres after rituximab treatment, durability of serological responses, recurrent thrombosis and safety in patients with APS.

**Methods:**

This retrospective study included 54 patients, predominantly with SLE-associated APS (*n* = 43), who received at least one course of rituximab. aCL and anti-β2-glycoprotein I (aβ2GPI) IgG/IgM were evaluated at baseline and 6–12 months after treatment. Long-term serological, clinical and safety outcomes were analysed over a median follow-up period of 4.7 years.

**Results:**

At 6–12 months, positivity rates decreased significantly for aCL IgG (59.3–24.1%), aCL IgM (24.1–5.6%), aβ2GPI IgG (42.6–16.7%) and aβ2GPI IgM (20.4–5.6%) (all *P* < 0.05). Long-term serological data were available for 31 patients. At last follow-up, aβ2GPI IgG positivity remained lower than baseline (*P* = 0.039), whereas quantitative titres were no longer significantly different. Long-term serological changes did not differ according to rituximab cycle number. Thrombotic recurrence occurred in five patients, four of whom were either off anticoagulation or had sub-therapeutic international normalized ratio values. Fifteen patients developed bacterial infections, seven SARS-CoV-2 infections and five patients died during follow-up, including one who died from sepsis.

**Conclusion:**

Rituximab was associated with marked short-term reductions in aPL titres and positivity, although quantitative suppression was not sustained long term. Changes in aCL/aβ2GPI did not consistently reflect thrombotic risk. These findings suggest that rituximab may be a valuable adjunctive therapy in selected patients with difficult-to-treat APS, although causality cannot be established from this retrospective study.

*Rheumatology* key messages

- Rituximab significantly reduced aPL titres and positivity rates within 6–12 months.
- Long-term serological suppression waned, despite persistently lower aβ2GPI IgG positivity.
- Changes in aCL/aβ2GPI were not a reliable predictor of residual thrombotic risk.

## Introduction

APS is a systemic autoimmune disorder characterized mainly by vascular thrombosis (arterial, venous or microvascular) and/or pregnancy morbidity, occurring in the persistent presence of aPLs, namely, LA, aCLs and anti-β2-glycoprotein I antibodies (aβ2GPI) [1]. While APS can occur as a separate entity, known as primary APS (PAPS), it may be associated with other systemic autoimmune diseases, most notably SLE.

The current standard of care for thrombotic APS is primarily long-term anticoagulation, often involving vitamin K antagonists (VKAs) [2]. However, despite adequate antithrombotic treatment, a subset of patients experiences recurrent thrombotic events, and anticoagulants do not address non-thrombotic manifestations of the syndrome, such as thrombocytopenia, haemolytic anaemia or heart valve disease [3]. Furthermore, anticoagulation targets the end step of the disease process rather than the initial steps such as B cell activation and the production of pathogenic autoantibodies [4]. Conceivably, there is an unmet need for immunomodulatory therapies that can modify the course of the disease, particularly for patients with refractory disease or contraindications to standard anticoagulation.

Rituximab, a chimeric monoclonal antibody that targets CD20-positive B cells, has emerged as a potential therapeutic option for refractory APS [5]. Theoretically, rituximab may reduce the precursor pool responsible for aPL production by depleting B cells, thereby lowering antibody titres and mitigating thrombotic risk. It has been shown to be effective in the management of catastrophic APS (CAPS), especially for patients who are refractory to standard therapy involving anticoagulation, glucocorticoids and plasma exchange/ IVIG [2, 6]. However, data regarding its specific impact on aPL serology and long-term clinical outcomes in chronic thrombotic APS remain heterogeneous, with findings depending largely on case series and pilot studies [7–10].

In this retrospective study, our aim was to evaluate the clinical and serological outcomes of APS patients receiving rituximab at a tertiary referral centre with a dedicated outpatient clinic for patients with SLE and/or APS. We assessed the short- and long-term dynamics of aPL, the incidence of recurrent thrombosis and adverse pregnancy outcomes, and the safety of rituximab.

## Methods

### Study population

This retrospective study included 54 consecutive patients with APS (±SLE) who received at least one cycle of rituximab (1000 mg on days 0 and 15) for any clinical indication and were monitored for at least 1 year after treatment at the SLE/APS outpatient clinic at Istanbul Faculty of Medicine between 2009 and 2024. There was no predefined operational definition of refractory APS and treatment decisions were based on individual clinical manifestations of APS and/or SLE. All patients with APS fulfilled the 2006 Sydney Classification Criteria, and all patients with SLE were classified according to the 2012 Systemic Lupus International Collaborating Clinics Classification Criteria [1, 11]. All data regarding the patients’ demographics, clinical characteristics, laboratory results, recurrent thrombotic and/or obstetric events, and mortality were retrieved from the database and revised. The duration of the disease was defined as the period between the initial diagnosis and the last visit or death, whichever occurred first. The study was conducted in accordance with the Declaration of Helsinki and was approved by the Clinical Research Ethics Committee of Istanbul Faculty of Medicine (approval number: 2024/715).

### Detection of aPL and follow-up after rituximab

The aPL profile included LA, aCL IgG/IgM and aβ2GPI IgG/IgM antibodies, with positivity confirmed on at least two occasions at least 12 weeks apart. aCL IgG/IgM and aβ2GPI IgG/IgM antibodies were detected using an ELISA (EUROIMMUN Diagnostics), with positivity accepted at a threshold of >40 GPL or MPL units or the 99th percentile. LA was measured using aPTT and dRVVT assays at the haematology laboratory, in accordance with the guidelines of the International Society on Thrombosis and Haemostasis [12]. Patients’ aPL profiles were evaluated prior to rituximab treatment (baseline) and then again 6–12 months after treatment. Since most patients were on anticoagulant therapy during and after rituximab treatment, LA positivity was not assessed at any point. However, the frequency of LA positivity at diagnosis is presented.

The evaluated outcomes included changes in aCL and aβ2GPI titres, as well as changes in positivity rates, at 6–12 months after treatment and at the last available long-term follow-up assessment. Repeat aPL testing beyond 12 months was not protocol-mandated and was performed at the treating physician’s discretion as part of routine clinical care. These analyses were compared with baseline and were also stratified according to the number of rituximab cycles received during follow-up (one vs more than one). We also evaluated the rate and details of thrombotic recurrence, mortality rates and the incidence of treatment-related adverse events, including hypogammaglobulinemia, infections and allergic reactions. Hypogammaglobulinemia was defined as serum IgG levels <700 mg/dl. The status and effectiveness of anticoagulant therapy during thrombotic recurrence were also evaluated.

### Statistical analysis

Statistical analysis was performed using IBM SPSS Statistics v.26 (IBM Corp., Armonk, NY, USA). Continuous variables were expressed as the mean ± standard deviation (S.D.) or the median (interquartile range [IQR]), depending on the distribution. Categorical variables were presented as frequencies and percentages. Comparisons of aPL titres before and 6–12 months after treatment were analysed using the Wilcoxon signed-rank test, while changes in the proportion of aPL positivity were analysed using McNemar’s test. For quantitative analyses, samples reported as negative without an available numerical titre were assigned a value of zero. A subgroup analysis was performed to evaluate the long-term durability of the serological response. This analysis included patients for whom aPL testing was available beyond 12 months after initial treatment. In this subgroup, the rates of aPL positivity and antibody titres at the last available follow-up visit were compared with baseline values. The incidence of thrombotic recurrence was calculated among patients with previous vascular thrombosis from first rituximab treatment to first recurrence, death or last follow-up, and expressed per 100 patient-years with exact 95% CIs. Statistical significance was set at *P* < 0.05.

## Results

### General characteristics of the patients

The study included 54 patients with APS, 11 (20.4%) of whom had PAPS and 43 (79.6%) had SLE and APS. The majority of the cohort were female (83.3%, *n *= 45). The mean age at diagnosis and median duration of disease were 30.2 ± 10.6 and 13.2 (IQR: 7.1–18.5) years, respectively. Vascular thrombosis was present in 43 patients, while 11 patients had isolated obstetric APS. The baseline clinical characteristics and aPL serology at the diagnosis of the patients are presented in Table 1. A descriptive comparison of the baseline characteristics of patients with PAPS and SLE-associated APS is provided in Supplementary Table S1.

**Table 1:** Clinical characteristics of the cohort and aPL serology at the diagnosis (*n *= 54).

Recurrent thrombosis, *n* (%)	28 (51.9)	Pregnancy morbidity, *n* (%)^a^	15 (33.3)
Venous thrombosis, *n* (%)	28 (51.9)	<16 w, ≥3 abortus	10 (22.2)
Deep vein thrombosis	24 (44.4)	16–34 w, ≥1 fetal death	8 (17.8)
Pulmonary embolism	7 (13)	Preeclampsia	3 (6.7)
Budd–Chiari syndrome	6 (11.1)	Placental insufficiency	1 (2.2)
Renal vein thrombosis	1 (1.9)	Non-thrombotic manifestations, *n* (%)	
Portal vein thrombosis	1 (1.9)	Heart valve disease	34 (63)
Venous sinus thrombosis	2 (3.7)	Thrombocytopenia	18 (33.3)
Arterial thrombosis, *n* (%)	28 (51.9)	Autoimmune haemolytic anaemia	11 (20.4)
Stroke	27 (50)	Livedo reticularis	10 (18.5)
Myocardial infarction	1 (1.9)	aPL serology, *n* (%)	
Digital ischaemia	2 (3.7)	LA	44 (81.5)
Microvascular thrombosis, *n* (%)	16 (29.6)	aCL IgG	33 (61.1)
Livedo racemosa	2 (3.7)	aCL IgM	14 (25.9)
Livedoid vasculopathy	3 (5.6)	aβ2GPI IgG	22 (40.7)
aPL-nephropathy	10 (18.5)	aβ2GPI IgM	13 (24.1)
Pulmonary haemorrhage	6 (11.1)	Triple aPL positive	21 (38.9)
Myocardial disease	3 (5.6)		

Values are presented as *n* (%) unless otherwise specified.

a The ratio among 45 female patients.

### Details of rituximab treatment and outcomes

Since rituximab could be administered for more than one indication in the same patient, treatment indications were not mutually exclusive. These were thrombotic APS (*n* = 28), LN (*n* = 10), thrombocytopenia (*n* = 10), musculoskeletal involvement (*n* = 8), neuropsychiatric lupus (*n* = 4), cutaneous lupus (*n* = 3) and autoimmune haemolytic anaemia (*n* = 1). Thrombotic APS was the sole recorded indication in 22 of these 28 patients. Among the six with overlapping indications, five also received rituximab for thrombocytopenia, while one had additional musculoskeletal and cutaneous indications. Of these 28 patients, 22 had experienced recurrent thrombosis and three had microvascular manifestations prior to treatment. The median follow-up period after rituximab treatment was 4.7 (IQR: 2.5–8.9) years. At least one additional course of rituximab therapy (500–1000 mg on days 0 and 15) was administered to 29 patients (53.7%) at least 6 months after the first course. Of the patients receiving rituximab treatment, 39 (72.2%) were also taking hydroxychloroquine, 30 (55.6%) glucocorticoids and 29 (53.7%) an adjunctive immunosuppressant, most commonly MMF (*n* = 21).

Six to 12 months after treatment, the proportion of patients testing positive for aPL had significantly reduced compared with baseline. Positivity rates decreased for aCL IgG (59.3% vs 24.1%, *P* < 0.001), aCL IgM (24.1% vs 5.6%, *P* = 0.020), aβ2GPI IgG (42.6% vs 16.7%, *P* < 0.001) and aβ2GPI IgM (20.4% vs 5.6%, *P* = 0.021). Quantitative analysis confirmed a statistically significant decrease in antibody titres across all tested isotypes (Fig. 1). In an exploratory subgroup analysis, seronegativization rates were higher, albeit not statistically significant, in patients with SLE-associated APS than in those with PAPS across all aPL isotypes (see Supplementary Table S1).

**Figure 1:**
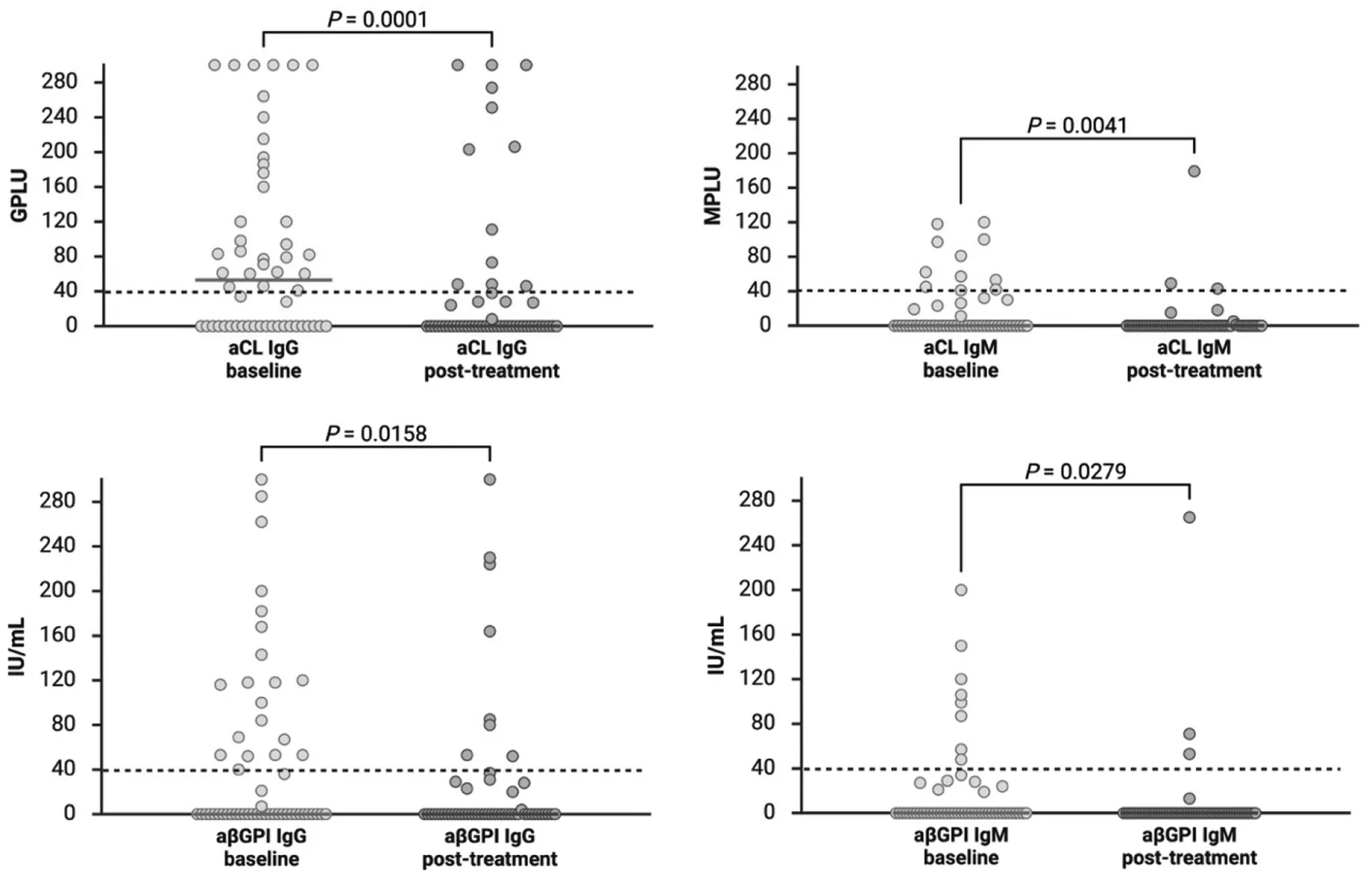
Change in aPL titres 6–12 months after rituximab treatment. Statistical significance was determined using the Wilcoxon signed-rank test. (Created in https://BioRender.com)

Long-term aPL antibody data were available for 31 patients, with a mean follow-up duration of 54.4 ± 28.8 months (range 14–111 months) after rituximab treatment. A comparison of the baseline characteristics and rituximab indications of patients with and without long-term serological follow-up is presented in Supplementary Table S2. Although the groups were broadly comparable, pre-rituximab aCL IgG positivity was less frequent among patients with long-term serological follow-up (45.2% vs 78.3%, *P* = 0.024). In 31 patients with long-term serological follow-up, aβ2GPI IgG positivity decreased significantly from 35.5% to 12.9% (*P* = 0.039). Reductions were also observed in the other isotypes, although these did not reach statistical significance. Specifically, aCL IgG positivity decreased from 45.2% to 32.3% (*P* = 0.388), aCL IgM from 16.1% to 6.5% (*P* = 0.375) and aβ2GPI IgM from 16.1% to 9.7% (*P* = 0.687). Among these 31 patients, 12 (38.7%) became completely seronegative at last follow-up. A quantitative analysis using the Wilcoxon signed-rank test showed that, at long-term follow-up, antibody titres across all tested isotypes did not differ significantly from baseline values (aCL IgG *P* = 0.501, aCL IgM *P* = 0.490, aβ2GPI IgG *P* = 0.976, aβ2GPI IgM *P* = 0.798). Detailed titre distributions at baseline, 6–12 months and long-term follow-up are presented in Supplementary Table S3. In exploratory, stratified analysis, 15 patients received 1 cycle of rituximab, while 16 received more than one. There were no significant differences in long-term changes in antibody titres from baseline between the 1 cycle and >1 cycle groups for any isotype (median change, aCL IgG, −46.0 vs −88.5, *P* = 0.463; aCL IgM, −25.0 vs −46.0, *P* = 0.643; aβ2GPI IgG, −35.0 vs −63.0, *P* = 0.648; aβ2GPI IgM, −18.0 vs −25.2, *P* = 0.714). Likewise, no significant between-group differences were observed in long-term changes in seropositivity. Among patients who were positive at baseline, seronegativity at last follow-up occurred in 4/6 vs 4/8 for aCL IgG (*P* = 0.627), 4/5 vs 4/6 for aβ2GPI IgG (*P* = 1.000) and 1/1 vs 3/4 for aβ2GPI IgM (*P* = 1.000) in the 1 cycle and >1 cycle groups, respectively. Overall, these findings suggest that, within the subgroup with available long-term serological data, long-term aPL changes were not significantly associated with receiving one vs more than one cycle of rituximab.

During the follow-up period after receiving rituximab, five recurrences occurred over 192.9 patient-years, corresponding to 2.59 events per 100 patient-years (95% CI, 0.84–6.05). Recurrence occurred in 4/28 (14.3%) patients with thrombotic APS as an indication for rituximab, and 1/15 (6.7%) treated for other indications (*P* = 0.643). Events occurred 10–81 months after the first rituximab course, after 1–3 treatment cycles (Table 2). Notably, two of the five patients were not receiving anticoagulant therapy at the time of recurrence. Of the three patients receiving VKAs, two had an international normalized ratio (INR) below the target range. Exploratory analyses according to triple aPL positivity, prior recurrent thrombosis and HCQ use showed no significant differences in thrombotic recurrence (Supplementary Table S4).

**Table 2:** Details of aPL-related events following rituximab treatment.

Patient	aPL profile at diagnosis	aPL titres (GPL/MPL) before RTX	aPL titres (GPL/MPL) after RTX (at last control)	Event	Number of RTX cycles	Post-RTX duration (months)	INR at event
1	Triple-positive	aCL IgG: 264, IgM: 0 aβ2GPI IgG: 0, IgM: 0	aCL IgG: 220, IgM: 145 aβ2GPI IgG: 227, IgM: 0	Pulmonary haemorrhage	3	38	2.4
2	Triple-positive	aCL IgG: 194, IgM: 0 aβ2GPI IgG: 100, IgM: 0	aCL IgG : 164, IgM: 0 aβ2GPI IgG: 195, IgM: 0	Myocardial infarction	2	24	1.4
3^a^	LA only	aCL IgG: 0, IgM: 0 aβ2GPI IgG: 0, IgM: 0	aCL IgG: 0, IgM: 0 aβ2GPI IgG: 0, IgM: 0	Stroke	1	19	Use only aspirin
4	LA + aCL IgG	aCL IgG: 86, IgM: 23 aβ2GPI IgG: 0, IgM: 0	aCL IgG: 5, IgM: 3 aβ2GPI IgG: 0, IgM: 0	Recurrence of digital ischaemia	1	10	1^b^
5	aCL IgM + aβ2GPI IgM	aCL IgG: 0, IgM: 120 aβ2GPI IgG: 0, IgM: 150	aCL IgG: 0, IgM: 9 aβ2GPI IgG: 0, IgM: 13	Skin necrosis	3	81	Use only aspirin

a Only lupus anticoagulant positive.

b An INR of 7.8 was reduced to 1 through the administration of vitamin K prior to the event due to vaginal bleeding.

Obstetric outcomes after rituximab were considered descriptive only, as only two patients conceived during the follow-up. One patient with isolated LA positivity at baseline had a spontaneous abortion before 10 weeks of gestation, 44 months after rituximab. The other patient who was triple aPL-positive prior to rituximab but negative for aCL IgG/IgM and aβ2GPI IgG/IgM before pregnancies, conceived 24 and 36 weeks after treatment. Both pregnancies ended in spontaneous abortions before 10 weeks of gestation.

Five patients died during the follow-up period after rituximab treatment. Their clinical characteristics are summarized in Table 3. The causes of death were complications of LN, treatment-refractory CAPS, sepsis, recurrent pulmonary haemorrhage, and traumatic intracranial haemorrhage. The time from rituximab treatment to death ranged from 6 to 81 months.

**Table 3:** Characteristics of patients who died during follow-up after rituximab treatment.

Patient	Diagnosis	aPL profile at diagnosis	aPL titres (GPL/MPL) before RTX	aPL titres (GPL/MPL) after RTX (at last control)	Cause of death	Number of RTX cycles	Post-RTX duration (months)
42/F	SLE + APS	LA + aCL IgG	aCL IgG: 60, IgM: 26 aβ2GPI IgG: 0, IgM: 0	aCL IgG: 0, IgM: 0 aβ2GPI IgG: 0, IgM: 0	LN	2	81
37/M	PAPS	Triple-positive	aCL IgG: 120, IgM: 10 aβ2GPI IgG: 200, IgM: 87	aCL IgG: 300, IgM: 12 aβ2GPI IgG: 300, IgM: 0	CAPS	1	6
32/M	SLE + APS	LA + aCL IgG	aCL IgG: 83, IgM: 0 aβ2GPI IgG: 0, IgM: 0	aCL IgG: 0, IgM: 0 aβ2GPI IgG: 0, IgM: 0	Sepsis^a^	1	29
34/M	PAPS	Triple-positive	aCL IgG: 264, IgM: 0 aβ2GPI IgG: 0, IgM: 0	aCL IgG: 220, IgM: 145 aβ2GPI IgG: 227, IgM: 0	Pulmonary haemorrhage	3	38
27/F^b^	SLE + APS	LA only	aCL IgG: 0, IgM: 0 aβ2GPI IgG: 0, IgM: 0	aCL IgG: 0, IgM: 0 aβ2GPI IgG: 0, IgM: 0	Traumatic intracranial haemorrhage	2	53

a IgG level at sixth month: 1790 mg/dl.

b Only lupus anticoagulant positive.

Infectious complications were observed in several patients. Bacterial infections occurred in 15 patients, four of whom required hospitalization. One fatal case occurred following ICU admission. Seven patients were diagnosed with a SARS-CoV-2 infection (two of whom required hospitalization), and three patients were diagnosed with herpes zoster. One patient hospitalized for SARS-CoV-2 had a serum IgG level of 479 mg/dl six months after rituximab, which increased to 700 mg/dl by the 12th month. Hypogammaglobulinemia was observed in an additional patient at month 6, which persisted through months 12 and 24. However, this patient did not develop any documented infections. In terms of infusion safety, two patients experienced allergic reactions, neither of which was considered as anaphylaxis.

## Discussion

In this retrospective study, we demonstrated that rituximab treatment resulted in a statistically significant reduction in the positivity rates and quantitative titres of aCL and aβ2GPI (IgG and IgM) within six to twelve months of therapy. Long-term follow-up revealed that, although aβ2GPI IgG positivity remained significantly lower than baseline levels, quantitative titres for all isotypes did not differ significantly from pre-treatment levels. In this predominantly SLE-associated APS cohort, we observed a low rate of thrombotic recurrence over a median follow-up of 4.7 years, suggesting that rituximab treatment may contribute to disease stabilization in selected patients with APS ± SLE. To the best of our knowledge, this study represents the largest single-centre experience regarding rituximab use in APS reported to date.

The serological response observed in our study is consistent with the pivotal role of B cells in APS pathogenesis. B cells may contribute to disease progression through antigen presentation and cytokine secretion, but primarily as precursors to plasma cells that produce pathogenic aPLs [5]. The short-term serological response to rituximab may therefore indicate the depletion of B cell subsets that replenish antibody-secreting cells that produce aPL, while long-lived plasma cells remain largely unaffected. B cell depletion may exert immunomodulatory effects by reducing cytokine production and antigen presentation. This could contribute to clinical effects independently of changes in aPL titres. The significant reduction in aPL titres observed in our study is consistent with the results of several retrospective studies that suggest B cell depletion can effectively reduce the burden of autoantibodies [7, 9, 13–15]. However, results of the RITAPS phase II descriptive pilot trial found no consistent change in aPL profiles following rituximab treatment, despite improvements in non-criteria clinical manifestations [10]. Similarly, a single-centre study conducted by Emmi *et al.* found no change in aPL titres despite a reduction in thrombotic relapse after rituximab treatment in seven SLE patients with APS [8]. We observed that, while antibody titres decreased significantly within the first six to twelve months, long-term suppression could not be achieved. Nevertheless, aβ2GPI IgG positivity rates remained significantly lower than baseline. Additionally, the numerically higher seronegativity rates observed in SLE-associated APS should be interpreted with caution, as aPL titres may fluctuate with SLE disease activity and therefore may not necessarily reflect a treatment specific effect. Some thrombotic events occurred despite reduced titres or solid phase seronegativity, whereas others occurred with persistent positivity. Longitudinal studies similarly suggest that aPL titres may fluctuate, and can decrease around thrombotic events, while persistent aPL positivity is associated with a higher risk of recurrence [16, 17]. Thus, changes in aCL and aβ2GPI alone may not reliably reflect residual thrombotic risk. While conventional aPL profiles help with risk stratification, they do not capture antibody fine specificity or functional pathogenicity. Differences in B cell dysregulation and the inflammatory milieu between PAPS and SLE-associated APS may also contribute to phenotypic heterogeneity and differential response to rituximab.

Rituximab has been shown to be highly effective in managing non-thrombotic manifestations of APS, particularly haematological involvement. Previous studies have reported high response rates and sustained remission in patients with refractory thrombocytopenia and autoimmune haemolytic anaemia following B cell depletion [18–20]. Additionally, the RITAPS trial demonstrated that patients experienced improvements in non-criteria manifestations, including skin ulcers, aPL nephropathy and heart valve disease [10]. Data from the “CAPS Registry” showed that 75% of the 20 patients receiving rituximab recovered from the acute catastrophic APS episode [6]. However, as most of these patients received a combination of therapies including anticoagulants, glucocorticoids, plasmapheresis and/or IVIG, either with or prior to rituximab, it is difficult to evaluate the effect of rituximab in isolation. Hence, rather than a first-line therapy in CAPS, recommended use is in refractory cases [2, 21]. In contrast to the evidence regarding non-criteria manifestations and CAPS, literature regarding the impact of rituximab in preventing recurrent thrombosis is scarce and heterogeneous [7–9, 15, 22–27]. Small rituximab series have reported absent or reduced thrombotic relapses during follow-up, although these studies had limited sample sizes [8, 9]. Although differences in patient characteristics, anticoagulation, and concomitant therapies preclude direct comparison, our recurrence incidence of 2.59 per 100 patient-years was numerically lower than rates reported in other observational APS cohorts (4.46–7.5 per 100 patient-years) [28, 29]. Importantly, inadequate anticoagulation was a major contributing factor in these cases; two patients were not receiving anticoagulation treatment at the time of the event and two others had sub-therapeutic INR levels. These findings support the usage of rituximab as a part of multi-target treatment rather than a replacement for standard anticoagulation therapy in patients at high risk of thrombosis.

In terms of obstetric outcomes, only one case of successful pregnancy has been reported in the literature following multidrug treatment including rituximab in a patient with refractory APS [30]. In our cohort, post-rituximab obstetric outcomes could not be meaningfully evaluated, as only two patients conceived during follow-up, resulting in three spontaneous abortions before 10 weeks of gestation. Given the very small number of pregnancies, and potential confounding by underlying APS risk, concomitant treatments, and timing of conception, no firm conclusions can be drawn regarding the efficacy or safety of rituximab in obstetric APS.

The safety profile observed in our study was generally consistent with the established risks associated with B cell depletion in systemic autoimmune diseases [10, 31]. Although infectious complications were observed, they should be interpreted cautiously in the context of a substantial underlying disease burden and concomitant immunosuppressive exposure, rather than being attributed solely to rituximab. Hypogammaglobulinemia was uncommon and not consistently associated with infection. Similarly, the fatal outcomes observed during follow-up appear to reflect not only infectious events, but also the severity of underlying diseases, including refractory LN, pulmonary haemorrhage, and CAPS. Notably, the patient with CAPS had a prior history of deep vein thrombosis without an established diagnosis of APS or long-term anticoagulant treatment, and initially presented to our centre with CAPS. Therefore, it is better to interpret this outcome as a manifestation of aggressive, previously unrecognized disease than as a treatment-related safety signal.

Our study has several limitations inherent to its retrospective design. Firstly, the study population was heterogeneous and predominantly consisted of patients with SLE-associated APS. Therefore, the results may not be fully generalizable to patients with PAPS. As aPL titres can fluctuate during the course of SLE, the reductions observed in our cohort may reflect the natural progression of the disease rather than being a specific effect of the treatment [32]. The small number of recurrent events also precluded reliable multivariable adjustment, and the stratified analyses should therefore be considered exploratory. Secondly, the lack of a control group, coupled with the fact that most patients were receiving concomitant medications, including HCQ, glucocorticoids and immunosuppressants, means that the efficacy of rituximab alone cannot be assessed directly, and comparisons with other therapeutic regimens are limited. Thirdly, long-term aPL testing was not protocol-based and was only available to a subset of patients, which could introduce selection bias. Historical data on assay changes and intra- and inter-assay variability were unavailable and may have affected longitudinal titre comparisons. Therefore, the long-term serological findings should be interpreted with caution and may not be representative of the entire cohort. Finally, a significant proportion of patients (53.7%) received at least one additional course of rituximab during the follow-up period, which could influence long-term serological and clinical outcomes. The exact timing of subsequent rituximab cycles and the data on B cell repopulation were unavailable, which limited the mechanistic interpretation. To minimize this confounding factor and isolate the effect of the induction therapy, we evaluated the initial decline in aPL titres 6–12 months after the first course of rituximab.

In conclusion, our data suggest that rituximab therapy effectively reduces aPL titres and positivity rates in the short term. However, this serological suppression tends to diminish over time and does not appear to differ with further rituximab cycles. Despite the predominantly relapsing nature of the disease, we observed a low rate of thrombotic recurrence, primarily due to inadequate anticoagulation rather than treatment failure. While the safety profile was manageable, the occurrence of fatal cases with infection and CAPS underscores the necessity for vigilant monitoring in this population. These findings suggest that rituximab may have a role as an adjunctive therapy in selected patients with difficult-to-treat APS manifestations, though future prospective studies are needed to define its clinical efficacy, optimal dosing strategies and long-term management protocols.

## Supplementary Material

keag609_Supplementary_Data

## Data Availability

The deidentified individual-level clinical and serological data supporting the findings of this study will be made available upon reasonable request to the corresponding author.
