# Supplementary material for: Serological and clinical outcomes of rituximab in APS with or without SLE: a retrospective single-centre cohort
Source: Rheumatology (Oxford). 2026 Oct 29;65(11):keag609. doi: 10.1093/rheumatology/keag609 (PMC13755874; doi:10.1093/rheumatology/keag609)
Supplement: Supplementary file 1 [file keag609_supplementary_data.docx]

**Supplementary Table S1** Comparison of baseline characteristics and outcomes between PAPS and SLE-associated APS

| **Characteristic** | **PAPS (n=11)** | **SLE-APS (n=43)** | **P value** |
| --- | --- | --- | --- |
| **Demographics** |  |  |  |
| Age at APS diagnosis, years, mean ± SD | 32.0 ± 6.4 | 29.7 ± 11.5 | 0.393 |
| Female, n (%) | 7 (63.6) | 38 (88.4) | 0.072 |
| **Clinical phenotype** |  |  |  |
| Vascular thrombosis, n (%) | 11 (100) | 32 (74.4) | 0.095 |
| Recurrent thrombosis before RTX, n (%) | 9 (81.8) | 19 (44.2) | **0.041** |
| Venous thrombosis, n (%) | 8 (72.7) | 20 (46.5) | 0.179 |
| Arterial thrombosis, n (%) | 9 (81.8) | 19 (44.2) | **0.041** |
| Microvascular thrombosis, n (%) | 6 (54.5) | 10 (23.3) | 0.065 |
| Pregnancy morbidity, n/N (%)* | 3/7 (42.9) | 12/38 (31.6) | 0.670 |
| **Non-thrombotic manifestations** |  |  |  |
| Cardiac valve disease, n (%) | 8 (72.7) | 26 (60.5) | 0.510 |
| Thrombocytopenia, n (%) | 2 (18.2) | 16 (37.2) | 0.301 |
| Autoimmune haemolytic anaemia, n (%) | 2 (18.2) | 9 (20.9) | 1.000 |
| Livedo reticularis, n (%) | 5 (45.5) | 5 (11.6) | **0.021** |
| **aPL profile at diagnosis** |  |  |  |
| Lupus anticoagulant positivity, n (%) | 10 (90.9) | 34 (79.1) | 0.667 |
| aCL IgG positivity, n (%) | 7 (63.6) | 26 (60.5) | 1.000 |
| aCL IgM positivity, n (%) | 3 (27.3) | 11 (25.6) | 1.000 |
| aβ2GPI IgG positivity, n (%) | 8 (72.7) | 14 (32.6) | **0.036** |
| aβ2GPI IgM positivity, n (%) | 4 (36.4) | 9 (20.9) | 0.429 |
| Triple aPL positivity, n (%) | 7 (63.6) | 14 (32.6) | 0.085 |
| **aPL profile before RTX** |  |  |  |
| aCL IgG positivity, n (%) | 9 (81.8) | 22 (51.2) | 0.092 |
| aCL IgM positivity, n (%) | 3 (27.3) | 10 (23.3) | 1.000 |
| aβ2GPI IgG positivity, n (%) | 8 (72.7) | 15 (34.9) | 0.039 |
| aβ2GPI IgM positivity, n (%) | 5 (45.5) | 6 (14.0) | 0.035 |
| **Clinical outcome after RTX** |  |  |  |
| New thrombotic or microvascular event, n (%) | 2 (18.2) | 3 (7.0) | 0.266 |
| **Serological outcome at 6–12 months**† |  |  |  |
| aCL IgG seronegativization | 3/9 (33.3) | **15/22 (68.2)** | 0.114 |
| aCL IgM seronegativization | 1/3 (33.3) | **7/10 (70.0)** | 0.510 |
| aβ2GPI IgG seronegativization | 3/8 (37.5) | **11/15 (73.3)** | 0.179 |
| aβ2GPI IgM seronegativization | 3/5 (60.0) | **4/6 (66.7)** | 1.000 |

* Pregnancy morbidity percentages are calculated among female patients.
† Seronegativization was defined as conversion from positivity before rituximab to negativity at 6–12 months

Categorical comparisons were performed using Fisher’s exact test; age was compared using Welch’s t-test. Analyses were exploratory and no adjustment for multiple comparisons was performed.

**Supplementary Table S2** Comparison of patients with and without long-term serological follow-up

| **Characteristic** | **Long-term serological follow-up (n=31)** | **No long-term serological follow-up (n=23)** | **P value** |
| --- | --- | --- | --- |
| **Demographics** |  |  |  |
| Age at APS diagnosis, years, mean ± SD | 29.3 ± 9.1 | 32.0 ± 12.5 | 0.382 |
| Female, n (%) | 26 (83.9) | 19 (82.6) | 1.000 |
| PAPS, n (%) | 6 (19.4) | 5 (21.7) | 1.000 |
| **Clinical phenotype** |  |  |  |
| Vascular thrombosis, n (%) | 25 (80.6) | 18 (78.3) | 1.000 |
| Recurrent thrombosis before RTX, n (%) | 16 (51.6) | 12 (52.2) | 1.000 |
| Venous thrombosis, n (%) | 16 (51.6) | 12 (52.2) | 1.000 |
| Arterial thrombosis, n (%) | 17 (54.8) | 11 (47.8) | 0.784 |
| Microvascular thrombosis, n (%) | 8 (25.8) | 8 (34.8) | 0.553 |
| Pregnancy morbidity, n/N (%)* | 9/26 (34.6) | 6/19 (31.6) | 1.000 |
| **Non-thrombotic manifestations** |  |  |  |
| Cardiac valve disease, n (%) | 23 (74.2) | 11 (47.8) | 0.086 |
| Thrombocytopenia, n (%) | 11 (35.5) | 7 (30.4) | 0.776 |
| Autoimmune haemolytic anaemia, n (%) | 6 (19.4) | 5 (21.7) | 1.000 |
| Livedo reticularis, n (%) | 4 (12.9) | 6 (26.1) | 0.294 |
| **aPL profile at diagnosis** |  |  |  |
| Lupus anticoagulant positivity, n (%) | 25 (80.6) | 19 (82.6) | 1.000 |
| aCL IgG positivity, n (%) | 16 (51.6) | 17 (73.9) | 0.158 |
| aCL IgM positivity, n (%) | 7 (22.6) | 7 (30.4) | 0.546 |
| aβ2GPI IgG positivity, n (%) | 10 (32.3) | 12 (52.2) | 0.170 |
| aβ2GPI IgM positivity, n (%) | 9 (29.0) | 4 (17.4) | 0.358 |
| Triple aPL positivity, n (%) | 9 (29.0) | 12 (52.2) | 0.099 |
| **aPL profile before RTX** |  |  |  |
| aCL IgG positivity, n (%) | 14 (45.2) | 18 (78.3) | **0.024** |
| aCL IgM positivity, n (%) | 5 (16.1) | 8 (34.8) | 0.197 |
| aβ2GPI IgG positivity, n (%) | 11 (35.5) | 12 (52.2) | 0.272 |
| aβ2GPI IgM positivity, n (%) | 5 (16.1) | 6 (26.1) | 0.498 |
| **Indications for rituximab†** |  |  |  |
| Thrombotic APS, n (%) | 16 (51.6) | 12 (52.2) | 1.000 |
| Lupus nephritis, n (%) | 5 (16.1) | 5 (21.7) | 0.728 |
| Thrombocytopenia, n (%) | 7 (22.6) | 3 (13.0) | 0.489 |
| Musculoskeletal involvement, n (%) | 3 (9.7) | 5 (21.7) | 0.264 |
| Neuropsychiatric lupus, n (%) | 4 (12.9) | 0 (0.0) | 0.127 |
| Cutaneous lupus, n (%) | 2 (6.5) | 1 (4.3) | 1.000 |
| Autoimmune haemolytic anaemia, n (%) | 0 (0.0) | 1 (4.3) | 0.426 |

* Pregnancy morbidity percentages were calculated among female patients.
† Rituximab indications were not mutually exclusive. Categorical comparisons were performed using Fisher’s exact test and age was compared using Welch’s t-test. Analyses were exploratory and no adjustment for multiple comparisons was performed.

**Supplementary Table S3** Distribution of aPL titres at baseline, 6–12 months, and long-term follow-up

| **Short-term paired titre distributions** | | | | |
| --- | --- | --- | --- | --- |
| **aPL** | **n** | **Baseline, median (IQR)** | **6–12 months, median (IQR)** | **P value** |
| aCL IgG | 54 | 61 (0–160) | 0 (0–38) | 0.0001 |
| aCL IgM | 54 | 0 (0–23) | 0 (0–0) | 0.0041 |
| aβ2GPI IgG | 54 | 0 (0–76.5) | 0 (0–21.5) | 0.0158 |
| aβ2GPI IgM | 54 | 0 (0–20) | 0 (0–0) | 0.0279 |
| **Long-term paired titre distributions** | | | | |
| **aPL** | **N** | **Baseline, median (IQR)** | **Long-term, median (IQR)** | **P value** |
| aCL IgG | 31 | 0 (0–92) | 5.0 (0.5–53.5) | 0.501 |
| aCL IgM | 31 | 0 (0–5.5) | 0 (0–2.25) | 0.490 |
| aβ2GPI IgG | 31 | 0 (0–53) | 1.5 (0–13.5) | 0.976 |
| aβ2GPI IgM | 31 | 0 (0–0) | 0 (0–4.65) | 0.798 |

Values are presented as the median (IQR). Samples with a qualitative negative result and no reported numerical titre were assigned a value of zero. P-values were calculated using the Wilcoxon signed-rank test. Short-term and long-term analyses included patients with paired data available at the relevant time points. Baseline distributions differ between panels because the paired populations differed according to follow-up availability.

**Supplementary Table S4** Exploratory analyses of post-rituximab thrombotic recurrence among patients with previous vascular thrombosis

| **Factor** | **Post-RTX thrombotic recurrence, n/N (%)** | **P value** |
| --- | --- | --- |
| Triple-positive | 2/19 (10.5) | 1.000 |
| Not triple-positive | 3/24 (12.5) |  |
| Prior recurrent thrombosis | 3/28 (10.7) | 1.000 |
| No prior recurrent thrombosis | 2/15 (13.3) |  |
| HCQ at rituximab | 5/33 (15.2) | 0.320 |
| No HCQ at rituximab | 0/10 (0) |  |

P values were calculated using Fisher's exact test. Analyses were restricted to patients with a history of vascular thrombosis (n = 43).
